# Spinal deformities rehabilitation - state of the art review

**DOI:** 10.1186/1748-7161-5-28

**Published:** 2010-12-24

**Authors:** Hans-Rudolf Weiss

**Affiliations:** 1 Orthopedic Rehabilitation Services, D-55457 Gensingen, Alzeyerstr. 23, Germany

## Abstract

**Background:**

Medical rehabilitation aims at an improvement in function, capacity and participation. For the rehabilitation of spinal deformities, the goal is to maintain function and prevent secondary symptoms in the short- and long-term. In patients with scoliosis, predictable signs and symptoms include pain and reduced pulmonary function.

**Materials and methods:**

A Pub Med review was completed in order to reveal substantial evidence for inpatient rehabilitation as performed in Germany. No evidence has been found in general to support claims for actual inpatient rehabilitation programmes as used today. Nevertheless, as there is some evidence that inpatient rehabilitation may be beneficial to patients with spinal deformities complicated by certain additional conditions, the body of evidence there is for conservative treatment of spinal deformities has been reviewed in order to allow suggestions for outpatient conservative treatment and inpatient rehabilitation.

**Discussion:**

Today, for both children and adolescents, we are able to offer intensive rehabilitation programmes lasting three to five days, which enable the patients to acquire the skills necessary to prevent postures fostering scoliosis in everyday life without missing too much of school teaching subjects at home. The secondary functional impairments adult scoliosis patients might have, as in the opinion of the author, still today require the time of 3-4 weeks in the clinical in-patient setting. Time to address psychosocial as well as somatic limitations, namely chronic pains and cardiorespiratory malfunction is needed to preserve the patients working capability in the long-term.

**Conclusion:**

Outpatient treatment/rehabilitation is sufficient for adolescents with spinal deformities.

Inpatient rehabilitation is recommended for patients with spinal deformities and pain or severe restrictive ventilation disorder.

## Background

Medical rehabilitation aims at an improvement in function, capacity and participation [1]. Outpatient and inpatient programmes are available worldwide for the rehabilitation of patients with impairments or disabilities in various medical fields. Particularly in Germany, there is a long history of inpatient rehabilitation for various diseases. The German Pension Insurance scheme has introduced a comprehensive practice guidelines programme for the development of process guidelines for inpatient rehabilitation. However, outcome studies in this field are very rare, which would justify the costs of such treatment. In the era of evidence based medicine, the usefulness, necessity and efficiency of inpatient rehabilitation has to be proven as does every other mode of treatment. For the German system of inpatient rehabilitation of chronic back pain, available evidence is not conclusive, due to a lack of randomised controlled studies. The prevailing design of observational cohort studies has severe limitations in proving a causal relationship between outcomes and intervention [2].

There are numerous papers providing evidence that outpatient rehabilitation is as effective as inpatient rehabilitation [3-10]. An outpatient cancer rehabilitation programme for instance, may be an effective alternative treatment to inpatient programmes for specific groups of patients [3].

There are no indications of poorer care quality in outpatient rehabilitation of orthopaedic patients, while economic analyses show better cost effectiveness in outpatient treatment by comparability of treatment, patients, and results [5]. The results of the latter study suggest that outpatient care, offered in the same quality as in the examined rehabilitation centres, is an alternative or complement to inpatient care at least for those patients, who can be treated in both the outpatient and inpatient setting [5]. Also different cardiac rehabilitation programmes (in- and outpatient) can be regarded as comparable concerning effectiveness and costs following rehabilitation [6].

### Limitations assumed in patients with spinal deformities

Rehabilitation is the process of assisting someone to improve and recover lost function after an event, illness or injury that has caused functional limitations. Rehabilitation engineering is the application of engineering sciences to design, develop, adapt, test, evaluate, apply, and distribute technological solutions to problems confronted by individuals with disabilities [1]. The term 'rehabilitation' however, includes psychosocial issues as well as participation and psychological stress.

But how can we define rehabilitation in the context of spinal deformities? First we need to acknowledge the functional limitations in patients with spinal deformities. An overview is given in the SOSORT consensus paper on physical exercises [11]:

For treatment of all spinal deformities, the goal is to maintain function and prevent symptoms in the short- and long-term. In patients with scoliosis, as summarized below, predictable signs and symptoms including pain and reduced pulmonary function may begin early in life and may worsen with age. Some curvatures still present at skeletal maturity also continue to worsen throughout life. For children with scoliosis, therefore, optimal treatment goals include reversing curvature magnitude and/or preventing curvature progression, pain, and pulmonary dysfunction over a lifetime.

#### *Pain*

Most clinical outcome surveys have revealed that, by early adulthood, the majority of scoliosis patients suffer from pain [12-26]. Only one large controlled survey has been carried out to date [27]. In that study, 1178 young adults, interviewed 10 years after diagnosis in adolescence, reported a significantly higher incidence of pain than 1217 control subjects. Of the scoliosis patients reporting pain, 23% (147/650) described it as *'horrible, excruciating, distressing' *compared with 1% (6/416) of the control subjects who reported pain. Similar results were reported at >44 year followup [28]. Of a subset of 69 patients treated in adolescence (from an original population of 444), twice as many scoliosis patients (77% vs 35%) suffered from pain compared with a population of adults of comparable age (> 55 years). Incidence of chronic pain was almost three fold higher in the scoliosis patients (61%) compared with the controls without scoliosis (22%). This is despite the fact that the 'control" popoulation was selected from hospital clinics, nursing homes, and senior citizens' centres where incidence of disability is exceptionally high [29,30]. How scoliosis causes pain is not clear, but the magnitude of pain in adult scoliosis patients recently has been found to be inversely proportional to curvature flexibility [31]. Related factors linked with pain include regional balance, instability and pathological mechanical loads on spinal elements [32].

#### *Pulmonary dysfunction*

Thoracic scoliosis in children results in characteristic signs of pulmonary dysfunction including reduced vital capacity (VC) and impaired exercise capacity (EC) [33-40]. Because the mechanism for impaired function is reduced, mobility of the chest wall deteriorates with age, pulmonary function deteriorates according to curvature magnitude even when the curvature itself does not progress [41-46]. In severe cases death occurs by respiratory failure [42-46]. The effects of reduced pulmonary function in patients with mild to moderate scoliosis are not known and have been dismissed as insignificant [e.g., [47,48]]. Recent studies, however, have shown that VC and EC characteristic of patients with mild to moderate scoliosis (< 85% predicted) are more reliable predictors of increased mortality than diabetes, high blood pressure, and heart disease [47-51]. Patient-described pulmonary symptoms, in general, are not a reliable indicator because patients are usually unaware of their limitations even when documented signs are severe and respiratory failure is imminent [40-44,51-55].

#### *Progression*

Once a flexible spinal curvature evolves into a spinal deformity, a 'vicious cycle' is initiated in which continuous asymmetric loading of the spinal elements fosters continued progression [55-57]. Only a few small surveys have examined the epidemiology of progression and insufficient information is available to reliably predict outcome for any given patient [58,59]. In general, the danger for dramatic progression is highest during periods of rapid growth, but most cases continue to progress throughout life [59-61]. Some individuals with similar curves exhibit marked progression after skeletal maturity while others are relatively stable [52]. The bases for such differences are unknown, though some have suggested that the likelihood of progression is greater the more rigid the curvature [62].

Taking into account the literature as listed above, scoliosis seems to coincide with a very bad prognosis if left untreated. On the other hand, reviews exist, which enlighten the prognosis of Adolescent Idiopathic Scoliosis (AIS) in another way [28,63]. Within these studies it has been shown that AIS is more benign than 'scoliosis' as investigated in other studies without differentiation of its etiology [28,63].

As AIS is the most common form of scoliosis, it seems appropriate to distinguish between this diagnosis and scoliosis of other origin.

Therefore, it seems reasonable to have a closer look at the early reviews by Weinstein [28] and a more recent review by Asher and Burton [63], clearly showing that the prognosis of AIS is not as bad as of scoliosis of other origin may be [28,63].

If we take into account the relative benign prognosis of AIS [28,63] the cost/effect relation of any therapy, conservative or operative, seems to be a major issue in times of restricted resources in the international health systems [64].

As has been pointed out by Hawes [65], scoliosis sometimes may have desastrous effects on the individual's health, however this is not necessarily the case for most scoliosis cases [28,63].

### Methodology of rehabilitation in patients with spinal deformities

Physical therapy and the application of spinal orthoses are the methods of choice as used in the context of rehabilitation in patients with spinal deformities. Additionally, psychologic support seems necessary by the specialist physician or is offered by psychologists.

### Role of exercise in rehabilitation of spinal deformities

**Figure 1:**
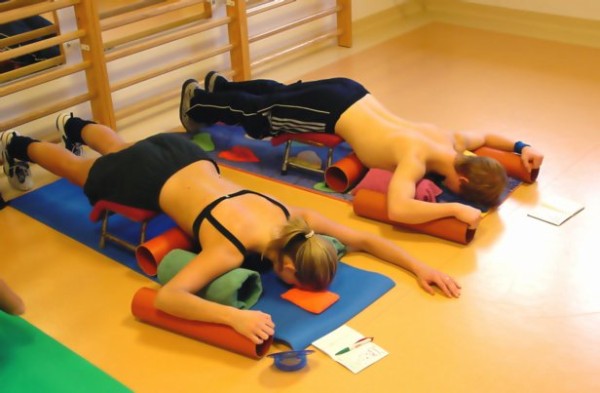
**Exercises as performed during inpatient rehabilitation**. Many items are needed in order to allow a pattern specific 3 D correction in lying position. Pads, stools, rolls and other items used in lying position are not needed in the new 'Power Schroth' approach used within the 'Best Practice' programme.

**Figure 2:**
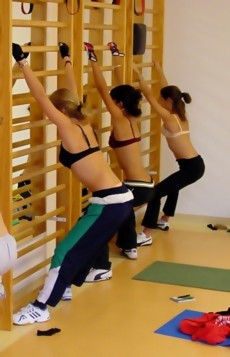
**Scoliosis is a 'flatback disorder' and therefore thoracic kyphosis has to be restored**. As shown in this example, many of the 'old' Schroth exercises increase the flatback. These exercises are still in use today, although this is not beneficial to AIS (Adolescent Idiopathic Scoliosis) patients with thoracic curvatures.

**Figure 3:**
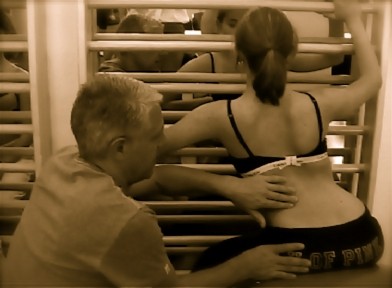
**New Power Schroth' exercise using postural synergy effects**. Outer rotation of the arm of the thoracic convex side improves derotation and redression of the rib-hump on the same side. Inner rotation of the arm of the thoracic concave side improves kyphosation on the thoracic concave side, which is the 'flatback side' of the curve.

Exercise based therapies (Figure 1, 2 and 3), alone or in combination with orthopaedic approaches, are a logical approach to improve and maintain flexibility and function in patients at risk for pain, pulmonary dysfunction, and progression. Published data reveal improved pulmonary function [54,66] and reduced pain [67,68] in response to scoliosis rehabilitation. Among the small number of studies which have examined this formally [69-76], progression was less in patient populations who were treated with exercise [reviewed in [77]]. When exercise was prescribed but not carried out by the patient, progression was similar to that of untreated populations [73].

The role of exercise based therapies as discussed in the spine literature has been controversial, however, with often repeated claims that research has shown that such approaches are ineffective in treating scoliosis [e.g. [78-90]]. An early systematic review of articles published in English throughout history produced no data in support of such claims [91]. As pointed out by Focarile et al., [92] in 1991, *'Experimental controlled studies of different therapies seem to be justified on both ethical and scientific grounds.'*

It was in 1941, when a committee of the American Orthopaedic Association undertook a study of methods and results of treatment of idiopathic scoliosis, by interviewing clinicians at sixteen clinics in the United States [93]. Case histories of 425 patients, followed for >1 year after treatment, were evaluated. The goal of the study was to 'establish the present status of this condition, and to clarify, as far as possible, what can be expected from the present methods of treatment.' Short term results obtained with surgery and with exercise were similar, with little or no improvement obtained for most patients.

In the ensuing decades since this study was published, the routine use of exercise for patients in the United States was largely eliminated. Meanwhile, an ongoing global effort to develop effective surgical methods is reflected in >10,000 peer reviewed articles published, in English, since 1950 and listed in Medline and other searches for scholarly articles. Unfortunately, the lack of success with exercise reported in 1941, unlike the failure of surgery, has not led to a corresponding effort to define improved methods for using physical therapy to treat patients with scoliosis: A parallel search of Medline revealed that fewer than 150 articles exploring the use of exercise-based approaches in the treatment of scoliosis in patients, of any age, have been published [11].

The routine use of exercise has remained central to therapeutic approaches in many countries [11]. To date, however, the body of literature available to patients and clinicians still seems of limited use [11,94]. The relatively limited literature in part reflects clinical traditions, which have not placed a high priority on publication. Perhaps more important, a diversity of approaches, standards, and languages limits how accessible and interpretable the available information is to colleagues with common interests [11]: Among several hundred reports of clinical outcome published in recent decades (> 600), no fewer than ten different languages were used. The establishment of a scientific society dedicated to research into scoliosis rehabilitation (SOSORT), and a venue for rigorous peer review of results from specialists, are critical first steps in defining the role of physical therapy in the treatment of scoliosis.

Nevertheless, in the latest updated reviews the use of exercises (Figure 3.) seems to have gained more evidence. When looking at scoliosis treatment including physical therapy, bracing and spinal fusion surgery, there is evidence on level II for conservative treatment and level III evidence, only for spinal fusion surgery [95-97].

In another systematic review Negrini and co-workers have found a randomized controlled trial for physical therapy in scoliosis management in Chinese literature. The authors claim from their findings, that physical therapy should be regarded as being of level I evidence [98].

The role of exercises in the treatment of kyphosis has never been investigated systematically, however, unlike exercises for the treatment of scoliosis, physical treatment of kyphosis patients seems to be accepted widely [99].

### The role of spinal orthoses in the rehabilitation of patients with spinal deformities

The use of spinal orthoses in the rehabilitation of patients with spinal deformities has been discussed controversially [94]. The evidence currently existent has been discussed in a comment [100] to the Cochrane review by Negrini and co-workers [101] as follows:

"Scoliosis is regarded as being a relatively rare condition and when using conservative measures, it cannot always be treated successfully. It would therefore seem likely that professionals accept such failures rather than search for reasons for this in conservative management. For example, Castro [102] claimed that it is necessary to achieve at least a 20% in-brace correction to stop curvature progression. In lieu of opening a discussion upon how to improve in-brace correction, the author accepted the failure and stated that, when a 20% correction cannot be achieved, brace treatment should be abandoned in favour of operative treatment.

**Figure 4:**
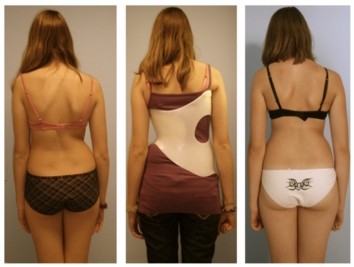
**Standard of today's high correction braces**. As can be seen on this figure, the deformity of the trunk is mirrored in the Gensingen brace™, one of the braces enabling correction of the spine and the clinical deviation of the trunk. After six weeks of bracing the deformity is largely reduced (right picture compared to left).

Whilst there is a wide range of braces available worldwide with many different approaches and theories proposed for each brace design, many of these braces lack evidence to support their use (Figure 4).

What we do know is: the outcome of brace treatment is determined by (1) in-brace correction effects and by (2) compliance of the patient treated [103]. We also know that risk for progression is highest during the pubertal growth spurt [104] and this knowledge has enabled the establishment of evidence-based guidelines for the treatment of scoliosis during growth [104,105]. Finally, we have to accept that while Randomized Controlled Trials (RCTs) have the highest evidence accepted [106], this study design is not appropriate to enable answers for all subsequent questions as has been discussed previously [106]. Neither patient characteristics, nor the very different braces in use today can be standardized enough to allow the application of a RCT.

Failures of > 40% have been reported in prospective studies [107,108], while in retrospective reports the failure rate has been < 10% [109-112]. Study designs included patients who, according to the SOSORT guidelines, did not necessarily need any treatment at all and whose authors claimed to have the smallest amount of patients requiring surgery [113]. Studies included patients from 4 to 14 years of age, with very few patients being at actual risk for progression [114]. The most progressive age range in girls with scoliosis is between 11 and 13 years, sometimes earlier. Therefore - also according to the SRS inclusion criteria - most of the population from the study cited would not have needed brace treatment at all. Nevertheless, the authors concluded that the intervention as described was beneficial [108,115], while other prospective controlled studies have included only patients at risk of progression leading to the conclusion that soft braces, presented in these studies [108,115], do not work at all [116,117]. Based on these study limitations, it is clear that more homogeneity within the outcome studies is needed to draw evidence-based conclusions.

An unbiased view on scoliosis treatment, not only rests in the study design, but also needs to include the consideration of many factors, determined by the patient samples and the different interventions used. Other factors include; age; gender; maturity; Cobb angle; rotation of the apical vertebra; curve patterns; curve stiffness; and compliance with treatment. The approaches to treatment are another important factor; brace type, specifics, and quality; in-brace correction; and also the appropriate approach to the patient to stimulate and maintain compliance with treatment.

The rarity of the condition, the many factors influencing the outcome of the intervention and the subjective and partly biased view of professionals, make it difficult to gain evidence for or against conservative or surgical treatment [97,118-121]."

For example, the Cochrane review on braces has been flawed for being incomplete. The authors did not include a prospective controlled trial with patient samples at actual risk for being progressive [100]. This study contains two homogenous groups of patients (girls, first signs of maturity), all premenarchial and fulfilling the SRS inclusion criteria for studies on bracing at least for the group of patients wearing the hard braces.

Interestingly, bracing in kyphosis patients has not been questioned to that extent [99], although there is much less evidence in international literature for kyphosis braces than for scoliosis braces [101].

**Figure 5:**
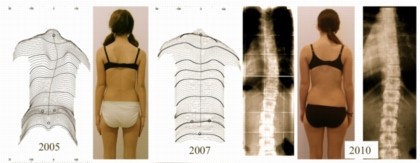
**Example of a patient with an initial overcorrection in a Chêneau light brace**. *Left *(2005) at begin with 38°, *middle *(2007) compensated appearance with 18° and finally *right *(2010) after weaning off (at 16 years of age) with a balanced clinical appearance the curve was 12° (right). Weiss and Werkmann *Scoliosis *2010 5:19 doi: 10.1186/1748-7161-5-1

Braces today are becoming more and more comfortable [122] and reliable with respect to in-brace correction effects [123-126] and seem to be able to also improve cosmesis (Figure 5)

### History of the rehabilitation of spinal deformities

Mechanical approaches have been used in ancient times. The table of Hippocrate (http://www.scoliosisjournal.com/content/4/1/6/figure/F18) and the suspension of scoliosis patients http://www.scoliosisjournal.com/content/4/1/6/figure/F14 have been used for many decades [127].

**Figure 6:**
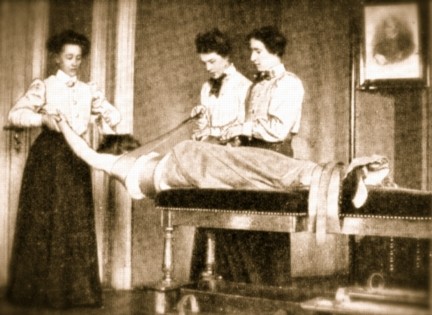
**Three therapists treat one patient in the setting proposed by Oldevig at the beginning of the 20th century**.

**Figure 7:**
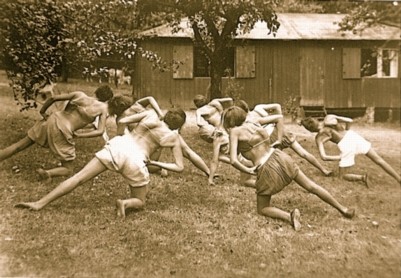
**Group rehabilitation therapy as proposed by Katharina Schroth**. The pattern specific programme has been taught to the patients so as to enable them to follow the instructions of the therapist during the group setting.

At the beginning of the 20^th ^century, different approaches of physiotherapy were used in the treatment of single patients (Figure 6), whilst since 1921, 3 to 6 months of rehabilitation in a group setting has been implemented by Katharina Schroth in Meißen, Germany [128,129]. There are case reports showing that with the Schroth approach, performed for some months, encouraging results have been achieved even in curvatures exceeding 100° Cobb (Figure 7).

In the 60's, 70's and 80's reasonable results were achieved and documented, even without the application of - at that time - braces of questionable quality. Case reports have been published in the book by Lehnert-Schroth, mother of the author and daughter of Katharina Schroth [129].

The rehabilitation of scoliosis patients has been subject of several investigations [66-68,70-72,75] and finally, it was possible to compare the results of a cohort treated between 1989 and 1991 followed up prospectively, to an untreated control group followed up prospectively from the same country [76]. The rate of progression has been reduced significantly in the group undergoing in-patient rehabilitation; however there still was a high proportion of patients with progression after in-patient rehabilitation.

While rehabilitation times have been reduced up to the end of the 90's [130], at the same time, the standard of bracing improved drastically in Germany. The development started in 1995 with new brace designs as implemented by Dr. Chêneau, who presented these at regular courses [126].

While the latest standards of specific braces show encouraging in-brace correction [123-126] and end results [123], in-patient rehabilitation with treatment times of 4 weeks or less and with programmes that have been widely changed presently seem to lack evidence [130].

As early as in 2006 it has been shown that rehabilitation time can be reduced significantly without further loss of outcome [131] and that the original programme can be easily improved by applying a new method to address the sagittal profile as an add-on [132].

Today, for both children and adolescents, we are able to offer intensive rehabilitation programmes lasting three to five days, which enable the patients to acquire the skills necessary to prevent postures fostering scoliosis in everyday life [133].

Nevertheless, in view of the fact that there are adolescent patients complying with bracing, who will have a beneficial outcome of treatment even without physiotherapy or in-patient rehabilitation, the issue of scoliosis rehabilitation will have to be analysed in more detail.

In times of reduced resources, the cheapest procedure of rehabilitation would be an appropriate brace worn according to the suggestions made in the SOSORT guidelines.

However, as long-term brace compliance is not easy to achieve, the supportive effect of group rehabilitation cannot be neglected [133].

Adults with scoliosis have been undergoing in-patient rehabilitation from the very start of such programmes [128,129]. There are in fact, many positive case reports showing successful outcomes after three months of rehabilitation in the little centre of Katharina Schroth in Meißen before World War II [129].

Contrary to adolescents or children with scoliosis, who have been shown to have beneficial outcomes after Scoliosis Short-term Rehabilitation (SSTR) [133], the secondary functional impairments adult scoliosis patients might have, in the author's opinion, even today need the time of 3-4 weeks in the clinical in-patient setting. Time to address psychosocial as well as somatic limitations, like chronic pain and cardiorespiratory malfunction is needed to keep the patients working, although final evidence for this is also lacking [130].

Nevertheless, the author has seen thousands of adult patients kept in work because of the positive impact of regular four-week rehabilitations with an intensive programme [134].

### Outcomes of spinal deformities rehabilitation

Today, there is no evidence that in-patient rehabilitation of patients with spinal deformities is superior to out-patient conservative management [130]. Although in the setting of in-patient rehabilitation it seems easier to reach the goals set with the help of multimodal approaches including psychological interventions, the outcome of today's in-patient rehabilitation does not seem superior to out-patient conservative management.

Retrospective studies show no difference between in- and out-patient management with respect to the surgical rate in children and adolescents with scoliosis. Surgical rates were roughly the same in a sample of patients treated as in-patients [111] as in three samples of patients treated in the out-patient mode [109,110,112].

A prospective study with a sample of patients undergoing in-patient rehabilitation of 6 weeks between 1989 and 1991, and a control group of patients not undergoing any treatment, reveals significant differences with respect to the progression rate [76], however neither length, nor intensity of the in-patient treatment is comparable to what is applied today [130].

A randomized controlled study as reported by Negrini and coworkers shows that out-patient physiotherapy in fact has a beneficial impact on patients with scoliosis in the mid-term [98], therefore the highest level of evidenced has been found for out-patient rehabilitation/physiotherapy.

Although the sample of patients in the study by Otman et al. [135] does not seem to be immature enough to draw final conclusions, the authors report improvements not only with respect to curvature, but also with respect to vital capacity, as has been shown in another study on in-patient rehabilitation [66].

Prospective controlled studies in a pre-/post design show, that scoliosis in-patient rehabilitation can be improved with specific add-ons and rehabilitation times can be reduced significantly without any loss of effectiveness [131,132].

Nevertheless, psychosocial issues may be better addressed in patients undergoing in-patient rehabilitation. However, evidence for this is weak [136,137].

There are studies demonstrating beneficial effects of in-patient rehabilitation in patient samples from the late 80's to the early 90's in patient samples undergoing treatment times of 6 weeks. Pain was reduced [67,68] and vital capacity significantly increased [66]. It has also been shown in a cohort study with a pre-/post-design that right cardiac strain was reduced after in-patient rehabilitation of 6 weeks.

Also curvature angles were improved during in-patient rehabilitation [71].

When reviewing literature, no long-term results of in-patient rehabilitation are available [130]. A prospective controlled study with adolescent patient samples from the years 1989 - 1991 has shown a beneficial mid-term effect of in-patient rehabilitation of 6 weeks compared to untreated controls [76].

For rehabilitation procedures applied today with reduced rehabilitation times and modified methodology no studies exist to support these interventions [130] and therefore it seems questionable as to whether the results mainly achieved in the years 1989 and 1991 can be achieved in todays setting of in-patient rehabilitation with reduced rehabilitation times, changed methodology and today's patient samples not being as motivated as in the 80's [76].

### Suggestions for a differential indication

#### Children and adolescents with spinal deformities

**Figure 8:**
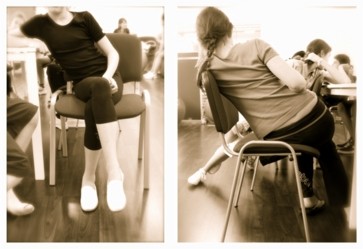
**Activities of daily living (ADL) as trained within modern Scoliosis Short-term Rehabilitation (SSTR)**. Corrected posture in standing, sitting and walking are essential to enable the patients to avoid postures in daily activities fostering curve progression. The SSTR is also taught to professionals regularily. Short impressions of the Scoliosis Short-Term Rehabilitation (SSTR) as described [133] can be found at: [144-146]. Impression of the courses given for professionals can be found here: [147,148].

**Figure 9:**
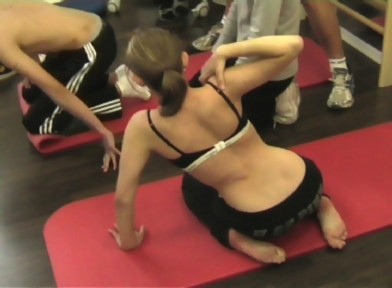
**A 'New Power Schroth' exercise to improve postural control of the patient treated**. This exercise, called ' Frog at the pond' enables the patient to achieve the skills necessary to improve postural control during ADL [146].

**Figure 10:**
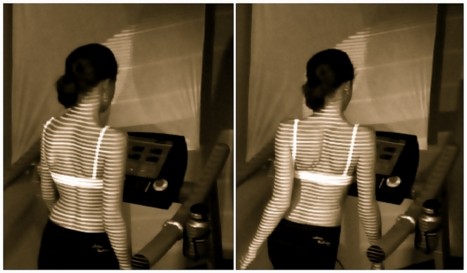
**Corrective movements trained during walking**. Scoliosis 3 D correction can be performed in sitting and also standing position, however the highest skill is correcting the deformity of the spine and trunk whilst walking. This can be performed using a treadmill, but also by walking plainly on the ground. This is also shown on a video [144].

Children and adolescents with spinal deformities without further limitations have to be treated according to the indication guidelines [104]. Overtreatment as well as undertreatment can be avoided when these guidelines are respected. In case there is an indication for physiotherapy, outpatient programmes are recommended (1) to avoid brace treatment or, in case of a brace indication (2) to support mobility of the spine as well as the compliance of the patients (Figure 8 and 9). In patients under brace treatment more intensive rehabilitation programmes are recommended [104], however these - according to current evidence - do not necessarily need to last a few weeks. Three to five day programmes have been tested successfully [133], but the author has also experienced beneficial outcomes of conservative treatment without any physiotherapy in patients with braces of best possible in-brace corrections. Brace treatment in the population with brace indication is surely the major issue and physiotherapy is secondary. Therefore, we should not overload our patients under brace treatment and with a good compliance with additional measures of questionable benefit. However, when rehabilitation of this special group of patients is indicated, the rehabilitation of daily activities - especially the rehabilitation of walking - is essential, as skills in corrective walking helps to gain the appropriate feeling for corrected posture in ADL (Figure 10).

The specialist guiding the patient has to have superior psychological skills, because a failure of brace treatment is not necessarily a brace failure, but in most cases fault of the physician [137].

#### Adolescents and adults with chronic back pains

Adolescents and adults with chronic back pains, require as well as physical treatment, psychological support [67,68,136,138]. Sometimes, the application of specific braces is also necessary [139,140]. Although there is no evidence that chronic pains can be treated more efficiently in the inpatient setting for patients with scoliosis and pain as to the authors opinion the safe environment of an inpatient centre should be chosen, because these patients have to cope with two different issues at the same time, deformity and pain, which require more complex approaches than for the rehabilitation of pain alone. The major aim of in-patient rehabilitation of adult patients with deformity pain is to preserve the patients working capability in the long-term.

Every inpatient centre specializing in pain management and with experience in scoliosis treatment will be able to address the problems and limitations of this specific group of patients accordingly [141].

#### Adolescents and adults with restrictive ventilation disorders

Adolescents and adults with restrictive ventilation disorders usually have severe thoracic deformities and lack rib mobility and vital capacity [11]. Both of these problems can only be addressed in specialized centres with superior knowledge in the field of rehabilitation of breathing. It has been demonstrated that vital capacity, rib mobility [66] and cardiopulmonary function [54] can be improved by undergoing an intensive inpatient rehabilitation programme lasting 6 weeks. While it is not clear as to whether 3 to 4 week programmes may have the same effect as the 6 week programme investigated in the 80's and 90's of the last century, inpatient rehabilitation should be performed in this group of patients as these patients have to cope with two different issues at the same time, deformity and restrictive ventilation disorder, which may require more complex approaches than for the rehabilitation of deformity alone.

#### Postoperative rehabilitation

Postoperative rehabilitation is rarely necessary. On the one hand direct postoperative mobility and activities of the patients' should be limited for a year in order to ensure a safe healing and stabilization of the bone structure. On the other hand, mobility of the spine as a whole and on the segmental level as well is largely reduced after operation. Problems within the fusion area cannot be addressed by physical means, manipulation of fused segments is obsolete and pain related to costotranversal joint problems can hardly be mobilized without stress to the fusion area.

Nevertheless, there is little evidence that operated patients with chronic back pain can benefit from inpatient rehabilitation [138]. The junctional zones (fused area/unfused area) of the spine can be stabilized and obviously pain can be reduced [138]. For the rehabilitation of operated patients with spinal deformities only specialized centres are recommended to assure maximum patient safety.

High quality rehabilitation with the help of exercises, re-education and high quality bracing may reduce the costs the community has to bear when surgical intervention can be avoided [142]. Finally it's the surgical intervention causing the highest costs after low quality conservative management has failed [142,143].

## Conclusions

There is little evidence that physiotherapy is beneficial in the treatment of patients with scoliosis.

There is some evidence that braces can stop curvature progression.

There is no evidence that in-patient rehabilitation today is superior to out patient conservative therapy.

Outpatient treatment/rehabilitation is sufficient for adolescents with spinal deformities without further limitations.

Inpatient rehabilitation is recommended for patients with spinal deformities and pain or severe restrictive ventilation disorder.

## Competing interests

The author is applying for some patents relating to the content of this paper and is advisor of Koob-Scolitech, Abtweiler, Germany.

## Consent

Written informed consent was obtained from the patients and their next of kin for publication of of their data within this review and accompanying images. A copy of the written consent is available for review by the Editor-in-Chief of this journal.
